# The impact of induced anxiety on response inhibition

**DOI:** 10.3389/fnhum.2013.00069

**Published:** 2013-03-07

**Authors:** Oliver J. Robinson, Marissa Krimsky, Christian Grillon

**Affiliations:** Section on the Neurobiology of Fear and Anxiety, National Institute of Mental HealthBethesda, MD, USA

**Keywords:** anxiety, threat, threat of shock, response inhibition, mind-wandering

## Abstract

Anxiety has wide reaching effects on cognition; evidenced most prominently by the “difficulties concentrating” seen in anxiety disorders, and by adaptive harm-avoidant behaviors adopted under threatening circumstances. Despite having critical implications for daily-living, the precise impact of anxiety on cognition is as yet poorly quantified. Here we attempt to clarify the impact of anxiety on sustained attention and response inhibition via a translational anxiety induction in healthy individuals (*N* = 22). Specifically, in a within-subjects design, participants completed the Sustained Attention to Response Task (SART) in which subjects withhold responses to infrequent no-go stimuli under threat of unpredictable electrical shock (anxious) and safe (non-anxious) conditions. Different studies have argued that this task measures either (1) attention lapses due to off-task thinking or (2) response inhibition; two cognitive functions which are likely impacted by anxiety. We show that threat of shock significantly reduces errors of commission on the no-go trials relative to the safe condition whilst having no effect on go trials or overall reaction time (RT). We suggest that this is because threat of shock during SART promotes response inhibition. In particular we argue that, by virtue of frequency, subjects acquire a habitual bias toward a go response which impairs no-go performance and that threat of shock improves the ability to withhold these prepotent responses. This improved response inhibition likely falls within the range of adaptive cognitive functions which promote cautious harm avoidance under threatening conditions, although a range of alternative explanations for this effect is discussed.

## Introduction

Anxiety can significantly alter cognitive function (Robinson et al., submitted). Prominent symptoms of anxiety disorders include attentional lapses and difficulty concentrating; sufferers often complain of an inability to stay focused on tasks because they are highly distractible. At the same time, in certain contexts—such as walking alone in the dark—anxiety can promote an adaptive state of improved vigilance and defense mobilization (Grillon and Charney, 2011). Whereas the effects of attentional capture by *acute* threat cues on cognitive performance is well-documented (e.g., threatening words alter performance on emotional Stroop tasks) (Algom, 2004; Pacheco-Unguetti et al., 2011; Padmala et al., 2011; Sagaspe et al., 2011; Pessoa et al., 2012), relatively little is known about the precise quantitative effects of more *sustained* anxiety states on cognitive and behavioral performance.

The present study examined the effect of sustained anxiety induced by unpredictable shock (Robinson et al., 2011; Cornwell et al., 2012) anticipation on performance of a go/no-go task designed to probe distraction (Robertson et al., 1997). In this so-called “sustained attention to response task” (SART), subjects are presented with frequent “go” stimuli, to which they have to respond, and infrequent “no-go” stimuli, to which they have to withhold responses (Robertson et al., 1997). This task was developed to measure lapses of attention and slips of action (i.e., off-task thinking) as indexed by errors of commission; e.g., inappropriate responses to the infrequent no-go trials (i.e., failed response inhibition). The impact of sustained anxiety on this task is, as yet, unknown.

Errors of commission on this task have been attributed to a number of different causes. One argument is that errors of commission represent “mind-wandering” or off-task thinking caused by boredom (Smallwood et al., 2004, 2009) and/or executive control failure (McVay and Kane, 2010). Mind-wandering involves relatively complex trains of thought which are primarily associated with the individual's current concerns (Klinger, 2009) and cause distraction from the task. However, this “mindless” theory of performance failure is not unanimously accepted. It is also argued that the task is a measure of response inhibition and impulsivity (Helton, 2008; Helton et al., 2009, 2010). Specifically, it is believed that the frequent go trials lead to a build-up of feed-forward, habitual, motor routines, which preserve task performance whilst reducing cognitive load. These responses are monitored by a supervisory system which controls “the strategic choices regarding the speed and accuracy of responses” (Helton et al., 2009). The supervisory attention system requires processing resources and can be weakened by cognitive load induced by task-relevant or -irrelevant thoughts, which leads to speeded reaction time (RT), increased RT variability, reduced response inhibition, and increased likelihood of errors of commission.

Anxiety induced by threat of shock has a wide range of effects on cognition (see Robinson et al., submitted), which leads to conflicting hypotheses regarding the impact of anxiety on this task. Anxiety could impair performance because it impairs executive control mechanisms that help maintain goal-directed behaviors (Bishop, 2009). It could also impair performance because threat of shock promotes lapses of attention and mind-wandering (e.g., off-task thinking) due to repetitive intrusive thoughts and worries (Watkins, 2008). This hypothesis is supported by reports that a lack of concentration in high state anxiety is correlated with mind-wandering (Watts and Sharrock, 1985) and with the observation that negative (i.e., sad) mood increases mind-wandering on the SART (Smallwood et al., 2007, 2009).

However, several lines of evidence point to the opposite hypothesis; that threat of shock should *reduce* errors of commission on SART. First, anxiety can facilitate perceptual/sensory processing (Robinson et al., submitted), which could lead to improved perception and detection of the infrequent no-go trials. Second, trait anxiety has been associated with enhanced response inhibition in go/no-go experiments (Sehlmeyer et al., 2010). Indeed, anxiety induced by threat of shock can increase inhibition of motor responses (Grillon and Davis, 2007; Cornwell et al., 2008). Specifically, prepulse inhibition, the mechanism by which a week sensory stimulus can, via temporal proximity, reduce eyeblink startle response to a loud noise, is *increased* by threat of shock. In particular, threat of shock serves to increase the ability of a weak acoustic or tactile “prepulse” stimulus to *gate* startle motor responding (Cornwell et al., 2008).

In this study, we therefore sought to discriminate between these conflicting possibilities and clarify the effects of anxiety on the SART. Subjects completed the SART task under conditions where they were at risk from-, and safe from, unpredictable shock. The main analysis focused on trial by trial RT and errors of commission, but we also examined RT to the trials that preceded no-go trials (Robertson et al., 1997) as errors of commission on no-go SART trials are commonly preceded by faster responding (Robertson et al., 1997). This has been argued to reflect an automatic mode of processing and off-task thinking (Robertson et al., 1997; Smallwood et al., 2004) but could also be interpreted as evidence of feed-forward prepotent, habitual motor response, and speed/accuracy trade-off (Helton, 2008). Finally, we examined self-report of off-task thinking (Smallwood et al., 2007) by asking subjects whether they were focusing on the task or if they experienced off-task thinking (anxious or otherwise).

Thus, a reading of the prior literature leads to conflicting hypotheses. On the one hand, anxiety could reduce the ability to maintain attention across trials through increased attentional lapses and anxiety-related thoughts manifested as increased RT variability and enhanced rates of errors of commission. On the other hand, anxiety could reduce errors of commission by improving sensory perception and/or response inhibition. Here, we aimed to distinguish between these two possibilities by examining the effect of anxiety induced by threat of shock on performance during SART.

## Methods

### Participants

Twenty two healthy volunteers (11 males, 11 females) between the ages of 20 and 34 (mean 27) were compensated for completing the study. Inclusion criteria were: (1) no past or current psychiatric disorders according to SCID-I/P (First et al., 2002), (2) no history of a psychiatric disorder in any first-degree relatives; (3) no medical condition that interfered with the objectives of the study as established by a physician, and (4) no use of illicit drugs or psychoactive medications according to history and confirmed by a negative urine screen. All participants gave written informed consent approved by the National Institute of Mental Health (NIMH) Human Investigation Review Board.

### Procedure

Following attachment of the electrodes, nine startle stimuli (habituation) were delivered every 18–25 s. This was followed by a shock work-up procedure to set up the shock intensity at a level highly annoying and mildly painful. Next, subjects performed a variant of SART (Robertson et al., 1997) when safe from shock and when anticipating shock.

### SART

Participants were asked to respond to frequent “go” stimuli (“=”) by pressing the space bar and to withdraw their response to rare “no-go” stimuli (“O”). These stimuli were randomly distributed and were presented for 250 ms at a rate of one every 2000 ms and there was no response deadline. There were a total of eight continuous 106-s SART blocks, four safe blocks, and four threat blocks that alternated. In each block, the go stimuli were presented on either 47 or 48 occasions while the no-go stimulus occurred four or five times per block for a total of 190 go and 18 no-go trials (adding up to 9.5% of total trials) per safe or threat condition. Three startle stimuli were delivered in each block to assess subjects' psychophysiological concomitants of anxiety during shock anticipation. The first SART block was a safe condition in half the subjects and it was a threat condition in the other half. Subjects were asked to give equal weight to speed and accuracy. A single shock was presented mid-block prior the final trial of two separate threat conditions, thus there were two shocks within a period of ~15 min; a sustained state of anxiety.

### Questionnaires

Subjective reports of on- and off-task thoughts as well as subjective anxiety were assessed after each block. Immediately after a block ended, subjects were asked to retrospectively rate their level of anxiety on a scale ranging from 1 (not at all anxious) to 10 (extremely anxious). On- and off-task thinking was evaluated by asking subjects about their thoughts at the time the block ended. They had to select one of the three choices indicating that they were (1) thinking about the task, (2) thinking about something unrelated (but not an anxious thought), or (3) having anxious thoughts. The sum total of each thought category was determined for each participant under each condition and the mean anxiety rating recorded for each condition.

### Stimulation and physiological responses

Stimulation and recording were controlled by a commercial system (Contact Precision Instruments, UK). The acoustic startle stimulus was a 40 ms duration 103-dB (A) burst of white noise presented through headphones. The eyeblink reflex was recorded with electrodes placed under the left eye. The electromyographic (EMG) signal was amplifier with bandwidth set to 30–500 Hz and digitized at a rate of 1000 Hz. The shock was administered on the left wrist.

### Data analysis

Following rectification and smoothing of the EMG signal, peak startle/eyeblink magnitude was determined in the 20–100-ms timeframe following stimulus onset relative to a 50-ms prestimulus baseline. The startle magnitude scores were averaged within the safe and the threat condition. Performance accuracy was determined for each condition (threat/safe) trial type (go/no-go) by dividing the number of correct trials by the total number of each trial type. The one trial following a shock was excluded from analyses. During the go condition, correct responses were any trial in which there was a response and in the no-go condition, correct trials are the ones in which no response was provided. RTs for correct go trials and incorrect no-go trials (errors of commission) were averaged across each condition. Response variability was determined by calculating the standard deviation in RT for (correct) go trials for each subject. To examine pre-error responses (Robertson et al., 1997), RTs were averaged across the four stimuli before no-go trials (Table 1), averaged across condition, and stratified by whether the subsequent no-go trial was or was not successful. The startle magnitude and subjective anxiety scores were averaged across blocks within each condition. Data were analyzed with repeated measures analyses of variance (ANOVA) and *T*-tests.

**Table 1:** **Behavioral measures; RT = reaction time (ms)**.

	**Threat**				**Safe**			
	**No-Go**		**Go**		**No-Go**		**Go**	
Accuracy	0.79	(0.05)	0.90	(0.02)	0.70	(0.06)	0.90	(0.02)
RT	370	(46)	361	(17)	295	(18)	359	(14)
	**No-Go**		**No-Go**		**No-Go**		**No-Go**	
	**fail**		**success**		**fail**		**success**	
Pre RT	293	(9)	357	(23)	316	(14)	364	(16)

## Results

### SART performance

**Figure 1:**
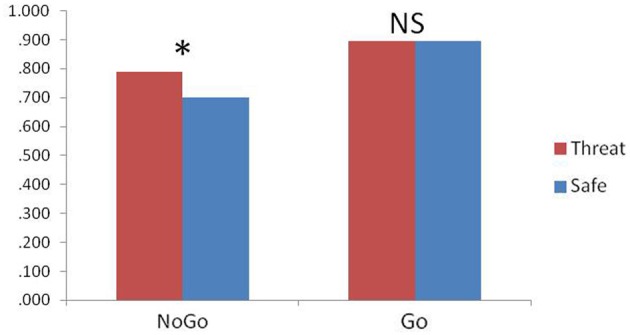
Response Accuracy; threat of shock significantly improved no-go accuracy (^*^p < 0.05), while having no effect upon go accuracy (NS = not significant), error bars represent standard error of the mean.

Accuracy was analyzed using a condition (safe, threat) × trial type (go, no-go) ANOVA. Consistent with previous results (Robertson et al., 1997), subjects were less accurate in responses to no-go trials compared to go trials [main effect of trial type; *F*_(1, 21)_ = 8.6, *p* = 0.008] as well as less accurate under safe relative to threat [*F*_(1, 21)_ = 4.7, *p* = 0.04]. However, accuracy to no-go trials was differently affected by the safe/threat conditions, leading to a significant condition × trial type interaction [*F*_(1, 21)_ = 8.9, *p* = 0.007]. The interaction was driven by a significant increase in no-go trial accuracy under threat relative to safe [*F*_(1, 21)_ = 6.8, *P* = 0.017; Figure 1]. Such a change in accuracy was not present for go trials [*F*_(1, 21)_ = 0.004, *P* = 0.9]. There was no significant difference in RT for correct go trials [*t*_(21)_ = 0.3, *P* = 0.8] or failed no-go trials [in which a response was recorded; *t*_(20)_ = 1.7, *P* = 0.1; note that this is a small number of trials so interpretation is limited; degrees of freedom in *t*-test is 20 because one subject had 100% accuracy]. RT variability was comparable across both safe and threat (SEM = 14 and 16 ms respectively) and a comparison of the standard deviation of each subject's go trials under safe and threat was not significant [*t*_(21)_ = −0.04, *p* = 0.97].

The pre-no-go trial RT were analyzed in a condition (safe, threat) × accuracy (fail, success) ANOVA. Results show a significant main effect of accuracy [*F*_(1, 18)_ = 15, *p* = 0.001] due to faster RT preceding failed compared to successful no-go responses that was not affected by the threat of shock {no condition × accuracy interaction; [*F*_(1, 18)_ = 0.4, *p* = 0.54]}. However, these results should also be treated with caution because they comprise a relatively small number of trials, particularly for failed no-go trials. Overall behavioral measures for each trial type and condition are presented in Table 1.

### Anxiety measures

There was a significant increase in state anxiety ratings under the threat (mean 5 ± 2) vs. safe (mean 2 ± 2) conditions [*t*_(21)_ = 6.8, *P* < 0.001]. This was associated with a comparable significant increase in raw startle response under threat (38) relative to safe (18) *t*_(21)_ = 3.3, *p* = 0.005.

### Probes of on-and off-task thinking

Following each block of the task, subjects thought equally about the task under threat and safe [*T*_(21)_ = 0.38, *p* = 0.7], but had more anxious thoughts under threat relative to safe [*T*_(21)_ = −3.2, *p* = 0.005], and more unrelated thoughts under safe relative to threat [*T*_(21)_ = 4.2, *p* < 0.001].

## Discussion

The main result of this study is that anxiety induced by threat of shock *reduced* errors of commission without affecting response speed or variability. These findings do not therefore support the hypothesis that threat of shock increased off-task thinking; as this would be expected to impair performance. Rather, we argue that induced-anxiety improved response inhibition.

We think that the most plausible explanation for better no-go accuracy during threat of shock is improved motor response inhibition. This is consistent with a number of different lines of research. Firstly, from a theoretical perspective, anxiety activates inhibitory behaviors. In fact, freezing is a well-established measure of anxiety (Gray and McNaughton, 2003). Secondly, event-related potential studies have suggested that trait anxiety is associated with enhanced motor response inhibition during no-go trials (Righi et al., 2009; Sehlmeyer et al., 2010). Thirdly, induced anxiety also increases prepulse inhibition of startle, that is, the ability to inhibit a startle motor response following a prepulse stimulus (Grillon and Davis, 2007; Cornwell et al., 2008). Indeed, the proportion of no-go trials in the SART is very low compared to the frequent go trials. As such, the task may be more a test of reactive stopping than proactive stopping (Aron, 2011). Specifically, by virtue of being more frequent, the go targets may acquire a bias toward habitual responding. Hence, no-go trials may be less about deciding *not to go* than *countermanding an initiated prepotent* response (Aron, 2011). Thus, anxiety may improve the ability to inhibit habitual responding. Such facilitation is of clear adaptive value as it may reduce the likelihood of an inappropriate motoric urge or impulsive response when threat looms.

The pattern of performance during SART could, however, potentially be due to the fact that anxiety facilitated detection of the no-go stimuli. Two potential mechanisms could lead to such an improvement: enhanced perception or focused attention. Substantial evidence shows that induced-anxiety facilitates perceptual/sensory processing (reviewed in Robinson et al., submitted). Such facilitation could help detect no-go trials. However, there is also evidence that anxiety increases the selectivity of attention. According to Easterbrook (1959)'s attentional breadth theory, anxiety narrows attention, reducing distraction by task-irrelevant peripheral stimuli. This view has been supported by several studies in which anxiety evoked by the anticipation of shocks leads to improved target detection (Agnew and Agnew, 1963; Tecce and Happ, 1964; Hu et al., 2012). Nevertheless, it seems unlikely that performance improvement under threat of shock was due to a better ability to detect or attend to stimuli on such a simple task with low perceptual load. First, several authors have noted that “Participants have no difficulty seeing and identifying the target” during SART (Cheyne et al., 2009; Helton et al., 2009). Second, as evidenced by the present data, participants only have difficulty withholding a response to the no-go trials; go trials, which are of equivalent perceptual demand, are uninfluenced. Third, on tasks specifically designed to probe vigilance, threat of shock actually serves to *impair* perception on high-load visual scanning tasks, whilst leaving low-load tasks intact (Cain et al., 2011). Thus, although firm conclusion must await further studies, it seems unlikely that the greater accuracy on no-go trials during threat of shock was driven by facilitated perception of or attention toward no-go stimuli.

Another possible explanation for improved performance during threat of shock is a non-specific increase in awakeness/arousal [i.e., alertness on a sleep wake spectrum (Oken et al., 2006)]. Anxiety increases arousal (Baas et al., 2006; Cornwell et al., 2007) and arousal can help maintain sustained attention (Oken et al., 2006). However, a key component of arousal is that it tends to decrease over time; and effect which is thought to underlie a phenomenon known as “vigilance decrement” (Helton, 2008; Warm et al., 2008; Helton et al., 2009). In particular, traditional sustained attention tasks are of long duration (longer than the SART) and require subjects to detect very rare targets. Such tasks are typically associated with a progressive decrement in performance thought to be driven, in turn, by progressively decreasing arousal (Helton, 2008; Helton et al., 2009). Errors of commission on the SART have, however, been shown to *decrease* over time when the test is repeated (Helton, 2008; Helton et al., 2009) which, if anything, would indicate *increasing* arousal as the task progressed. Helton and others have in fact argued that, rather than measuring sustained attention *per se*, performance on the SART reflects a strategic decision regarding speed/accuracy trade-off (Helton, 2008). This hypothesis is based on the observation that, over time, errors of commission go down while RT goes up (Helton, 2008). The present study, as well as previous studies (Robertson et al., 1997; Smallwood et al., 2004), provide further support for this speed accuracy trade-off argument by demonstrating that errors of commission are preceded by faster RT than non-errors. Thus, more cautious RT leads to greater accuracy. However, this effect does not vary across safe/threat conditions and hence unlikely explains the improved performance under threat. In other words, threat seems to improve accuracy at no cost to speed, providing no evidence for a speed/accuracy trade-off.

It should be noted that the effect seen here is distinct from that seen when discrete threatening or aversive cues are utilized in go/no-go tasks. For instance cues which have been paired with shocks as well as aversive faces [more analogous to “fear” than “anxiety” (Grillon, 2008)] serve to impair inhibitory control (Padmala et al., 2011; Sagaspe et al., 2011; Pessoa et al., 2012). Indeed, anxiety can impair inhibitory control in the context of affective targets in Stroop like paradigms (Pacheco-Unguetti et al., 2011). The key difference between these studies and the present study is that in the present task the stimuli are affectively neutral. Indeed, for the purposes of harm avoidance, it makes adaptive sense to allocate resources toward threatening stimuli in the context of anxiety (even at the expense of impaired inhibition). At the same time, it makes sense to improve the overall ability to inhibit responding in the absence of threatening stimuli. Thus, the overall behavior is likely the result of an interaction between the sustained state (anxiety), the valence of the stimuli being processed (e.g., aversive vs. neutral stimuli) and the motor response.

In summary, we present novel data demonstrating that anxiety induced by threat of shock can improve the ability to withhold responses to infrequent targets on a go/no-go task. We argue that this effect reflects facilitated inhibition of habitual motor responses, which may be a part of a broader pattern of anxiety improving cognitive and perceptual processes, perhaps for the sake of better improving harm avoidance (Robinson et al., submitted). It should be noted that errors of commission during SART have been typically used as evidence of mind-wandering (Robertson et al., 1997; Smallwood et al., 2004, 2009). However, errors of commission are only indirect measures of mind-wandering and can be affected by other processes, such as changes in perceptual processing or response inhibition. We believe that reduced errors of commission in the present study did not reflect reduced off-task thinking during threat of shock but better response inhibition, although we *also* believe SART may be useful to study off-task thinking and more specifically anxious thoughts. Future studies may attempt to use more comprehensive thought sampling methodologies (Smallwood and Schooler, 2006) to tap into subjective experiences, as well as attempt to clarify the neural substrates of this effect using fMRI and EEG, in both healthy and patient populations.

### Conflict of interest statement

The authors declare that the research was conducted in the absence of any commercial or financial relationships that could be construed as a potential conflict of interest.
